# Matching enrolled trial participants to disease demographics: Using IRB submissions to identify opportunities for researcher training

**DOI:** 10.1017/cts.2023.539

**Published:** 2023-05-08

**Authors:** Elizabeth Eckstrom, Meredith Zauflik, Bryanna De Lima

**Affiliations:** 1 Division of General Internal Medicine & Geriatrics, Department of Medicine, Oregon Health & Science University, Portland, OR, USA; 2 Oregon Clinical & Translational Research Institute, Oregon Health & Science University, Portland, OR, USA

**Keywords:** Disease demographics, older adults, lifespan, inclusion in research, trial recruitment

## Abstract

**Background:**

Many diseases are highly prevalent in older adults, yet older adults are often underrepresented in corresponding trials. Our objectives were to (1) determine alignment between Institutional Review Board (IRB) protocol age ranges and enrollment demographics to disease demographics pre- and post-implementation of the 2019 National Institutes of Health (NIH) Lifespan Policy and (2) raise awareness about inclusive recruitment to principal investigators (PIs).

**Methods:**

This was a pre-post study. We reviewed investigator-initiated studies meeting eligibility criteria at Oregon Health & Science University from 2017 to 2018 to determine baseline alignment. Alignment was defined by the level of matching between protocol/enrollment age and disease demographics: 2 points for full match, 1 point for partial match, and 0 points for mismatch. After the NIH policy implementation, we reviewed new studies for alignment. When a mismatch was determined, we contacted PIs (either at initial IRB protocol submission or during ongoing recruitment) to raise awareness and provide strategies to expand inclusion of older adults in their trials.

**Results:**

Studies that matched IRB protocol ages to disease demographics significantly improved from 78% pre-implementation to 91.2% post-implementation. Similarly, study enrollment ages matching disease demographics increased by 13.4% following the implementation (74.5%–87.9%). Out of 18 post-implementation mismatched studies, 7 PIs accepted a meeting and 3 subsequently changed their protocol age ranges.

**Conclusion:**

This study highlights strategies that translational institutes and academic institutions could use to identify research studies whose participants do not align with disease demographics, offering opportunities for researcher awareness and training to enhance inclusion.

## Introduction

In the USA today, 80% of dementia cases occur in people over the age of 80 years; however, 80% of dementia research involves participants under the age of 80 years [1]. Osteoporosis, heart disease, and many other diseases have similar discrepancies between the demographics of research participants and the demographics of the population most impacted, particularly for older adults [2,3]. To address such discrepancies, and to ensure the inclusion of older adults in research, the National Institutes of Health (NIH) implemented the “Inclusion Across the Lifespan” policy (NOT-OD-18-116) [4], which became mandatory in January 2019. This policy requires that investigators submitting human subjects applications to the NIH “address plans for including individuals across the lifespan…so that knowledge gained from NIH-funded research is applicable to all those affected by the researched diseases/conditions.”

Oregon Clinical and Translational Research Institute (OCTRI), the NIH Clinical and Translational Science Awardee hub at Oregon Health and Science University (OHSU), implemented an OHSU Institutional Review Board (IRB) approved study titled “OCTRI IRB Lifespan Inclusion Project” as part of their efforts to ensure that OHSU investigators follow the new NIH inclusion policy. The study goals were to (1) determine the level of alignment between study participant ages and disease demographics before and following implementation of the NIH Lifespan Inclusion Policy and (2) use these results to catalyze change in recruitment of older adults in all OHSU research studies by educating principal investigators (PIs).

## Materials and Methods

### Eligibility and Study Design

**Figure 1.**
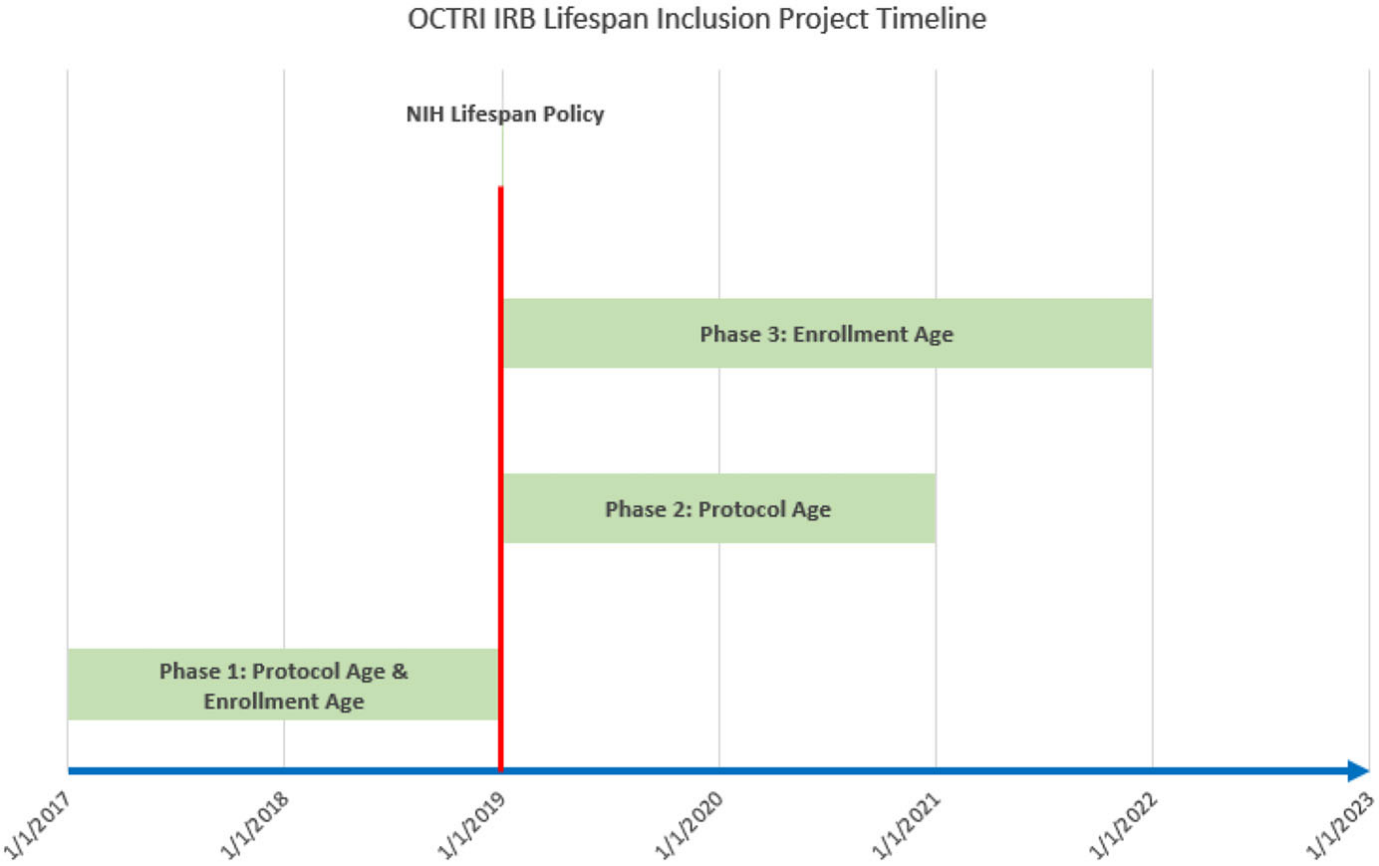
Timeline of phases throught this study. Red vertical line indicates the start of the NIH Lifespan Policy in January 2019. Phase 1 was the 2-year lookback period assessing protocol age and enrollment age against disease demographics and disease prevalence range. Phase 2 was a 2-year prospective period assessing protocol age against disease demographics and disease prevalence range. Phase 3 was a 3-year prospective period assessing enrollment age against disease demographics and disease prevalence range.

This study had three phases (Fig. 1). The first phase was retrospective and focused on the 2 years prior to implementation of the 2019 NIH Lifespan Policy. In order to be eligible for this review, studies had to be initiated by an OHSU investigator (defined as “PI involved in the design of the protocol”), require prospective consent, address a disease or condition relevant to older adults (defined as “individuals 65 + years old”), and be closed to enrollment between January 2017 and December 2018. Studies were screened for eligibility by review of IRB project title, proposed age range, and brief study description. Studies were excluded if they were industry-sponsored (unless the industry involvement was funding only and the study was investigator-initiated); if they were multicenter trials where OHSU was not the coordinating center; or if the disease in question was not relevant to older adults (e.g., maternal/neonatal diseases and pediatric cancers). We reviewed these studies to see if there was a mismatch between inclusion criteria age ranges and the age demographics of the studied disease or research question. Mismatch was determined based on review of the demographics of the disease or condition being studied compared to the demographics of the study as submitted. Demographics of diseases were determined through the use of Centers for Disease Control and Prevention [5], NIH [6], and other organizations that publish this information. We searched databases available from these entities to find the most comprehensive demographic information and then combined this information to create a disease demographic reference sheet (see Supplemental Materials). Each disease or condition was assigned both a disease demographics range (based on the highest and lowest age at which the condition typically occurs) and a prevalence range (based on the ages at which the condition is most prevalent).

The second phase of the study was prospective and evaluated all new submissions to the IRB for the 2 years following implementation of the NIH Lifespan Policy (dates 1/1/2019–12/31/2020) to compare the disease demographics to the protocol age criteria of studies. Eligibility included new studies submitted to the IRB that were PI-initiated, PI had control over the protocol (e.g., if Multi-site Clinical Trial were the Coordinating Center/Data Coordinating Center), disease-focused, diseases relevant to older adults, and that were submitted to the IRB after 1/1/2019 and prior to 12/31/2020. Interventions in this phase included reaching out to the PI if there was a “mismatch” between disease age range and protocol age range to discuss the rationale for their age range and offer support for adjusting their age range if appropriate. These meetings were conducted by one of the study authors (EE) and followed the protocol: (1) investigator was questioned to learn details of their research study (that could not be gleaned from the IRB proposal); (2) study author reviewed disease demographics with investigator and compared demographics to the IRB protocol age range; (3) study author asked if the investigator would like to expand their age range and offered suggestions for how to do that, including using electronic health record cohort discovery tools, local registries the investigator wasn’t aware of, and suggestions to adapt the protocol to be more feasible and accessible for older adults.

The third phase of this study was prospective monitoring of enrollment of new studies for 3 years after the NIH Lifespan Policy (dates 1/1/2019–12/31/2021) to compare the disease demographics to the enrollment age ranges of studies. Intervention in this phase included reaching out to the PI if there was a “mismatch” between disease age range and enrollment age range to provide tips for recruitment and retention of older adults. The reach out at this phase of the study was similar to the reach out in Phase 2, with added emphasis on retention of older adults.

### Data Collection and Analysis

OHSU IRB approval (#STUDY00018894) was received prior to collection of this data. A waiver of documentation of informed consent was approved for this study as the record review posed no more than minimal risk to individuals, appropriate subject privacy protection procedures were in place, and there was no interaction or intervention with the study participants. No sample size calculation was performed because all eligible studies were included.

Data to assess study eligibility were collected using OHSU’s clinical trial management system (“eCRIS”) and IRB records. Study-level information, including disease or condition being studied and inclusion and exclusion criteria, was collected for studies meeting eligibility criteria. Each study was summarized by the targeted disease and proposed minimum and maximum participant ages (referred to as protocol age range). Of note, the protocol age range was determined prior to study enrollment. Participant information – age, race, ethnicity, and gender – was used to summarize the number of total subjects, average participant age, minimum age, maximum age, and age distribution (in the form of a histogram). All study-level and participant-level data were maintained in a REDCap database.

**Figure 2.**
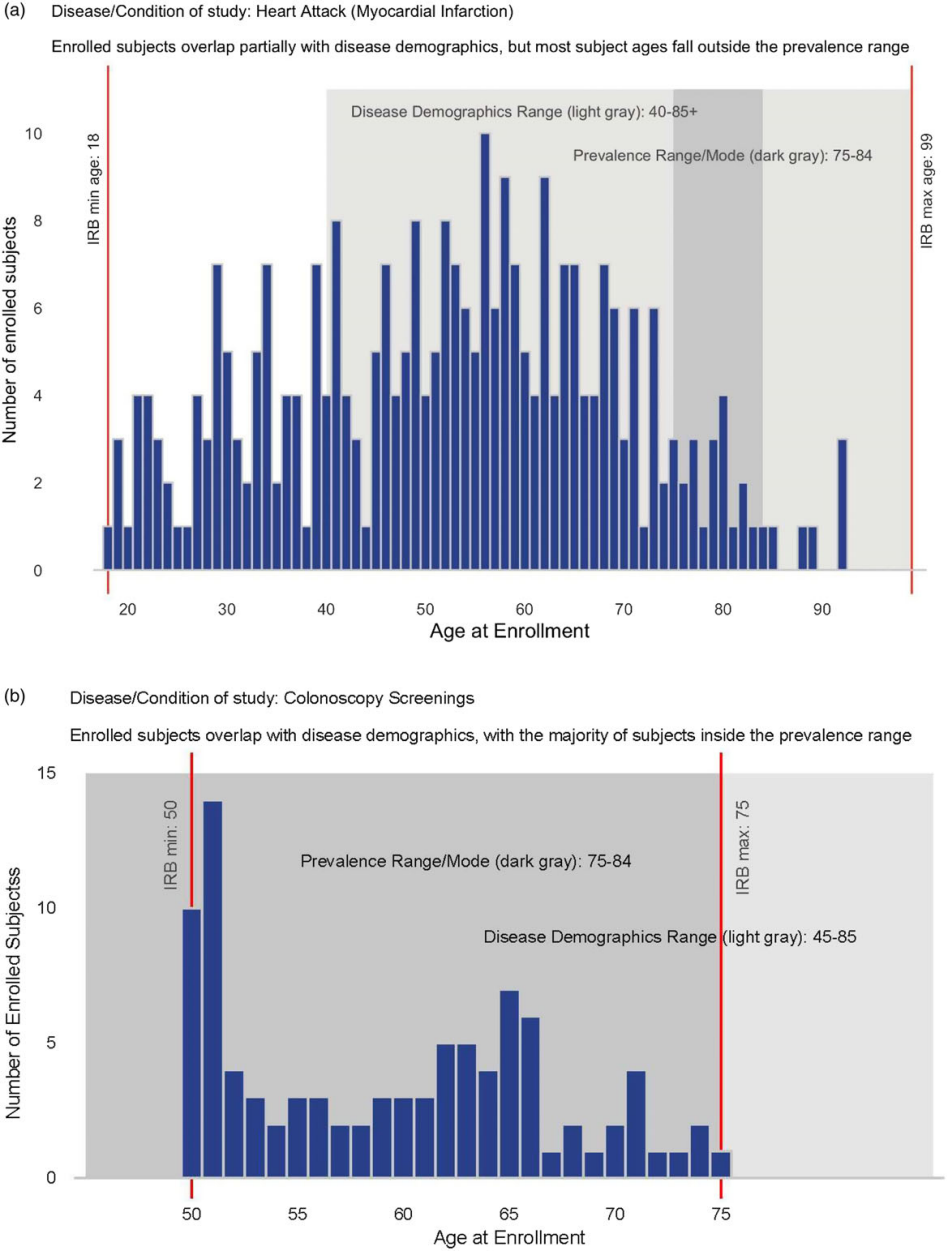
Histogram examples displaying the overlap of enrollment age against disease demographic range and disease prevalence range for a mismatch (***a***) and match (***b***). Disease demographics range, shown in light gray, is the highest and lowest age at which the condition typically occurs. Disease prevalence range, shown in dark gray, is the ages at which the condition is most prevalent.

For Phase 1, disease demographics and study data were reviewed and analyzed to determine the level of disease to population “match” or “mismatch.” Participant histograms were charted alongside the protocol minimum and maximum ages, disease demographics range, and prevalence range (Fig. 2) and could be used with research teams to show them how their study age ranges compared with disease age ranges. These charts were reviewed alongside study summaries by the lead author (EE) and scored on each of the following four criteria: (1) how well the protocol age range (as determined by the study team prior to submission to the IRB) matched the disease demographics range (defined as the highest and lowest age at which the condition typically occurs); (2) how well the protocol age range matched the disease prevalence range (defined as the most common ages at which the disease occurs); (3) how well the enrolled subject ages matched the disease demographics range; and (4) how well the enrolled subject ages matched the disease prevalence range. Studies that were a full match (i.e., age ranges matched completely) received two points. Those that were a partial match (i.e., age ranges overlapped but had some mismatch) received one point. Those that were a mismatch (i.e., age ranges mostly did not match) received 0 points. The scoring matrix for each criteria is shown in Table 1. We counted the number and percent of studies with discrepancies and determined which proposals were noncompliant with the NIH Lifespan Policy. We specifically determined if enrolled participants in OHSU studies (a) matched the demographics of the disease in question, (b) fell within the proposed age range for recruitment, and (c) were appropriately distributed across the proposed and/or adjusted age range (as mandated by the NIH Lifespan Policy).

**Table 1.** Scoring matrix used to evaluate matching between protocol age and enrollment age to disease demographics and disease prevalence range

Variable name	Description	2 = Mostly/ completely	1 = Somewhat	0 = Not at all
IRB Protocol Age – Disease Demographic Score	How well IRB min/max ages reflect disease demographics	Disease demographics range fits entirely within the IRB min/max ages	Disease demographics range fits partially within the IRB min/max ages	Disease demographics range is entirely outside the IRB min/max ages
IRB Protocol Age – Prevalence Score	How well IRB min/max ages reflect disease prevalence range	Prevalence range fits entirely within the IRB min/max ages	Prevalence range fits partially within the IRB min/max ages	Prevalence range is entirely outside the IRB min/max ages
Enrollment Age – Disease Demographic Score	How well enrolled subject ages reflect disease demographics	All/most participants are within the disease demographics range	Some participants are within the disease demographics range, but not most	No/very few participants are within the disease demographics range
Enrollment Age – Prevalence Score	How well enrolled subject ages reflect disease prevalence range	The mode of participants enrolled is within the prevalence range	Some participants are within the prevalence range, but the mode is elsewhere	No/very few participants are within the prevalence range

For Phase 2 of the study, all eligible new IRB submissions starting in January 2019 (when the NIH Lifespan Policy was implemented) were reviewed in 6-month blocks, and eligible age ranges were determined to be compliant or noncompliant with the policy. Studies in this phase were assessed on the first two criteria listed above related to protocol age: (1) how well the protocol age range matched the disease demographics range and (2) how well the protocol age range matched the disease prevalence range. Studies that were a full match for the two criteria were totaled separately, and the percentage was calculated for both. If a study was deemed to be noncompliant with the policy, our research team reached out to the PI of the study to request a meeting. In that meeting, the PI was asked their rationale for the choice of age range for their study, potential concerns about raising the upper age limit for inclusion in the study, and exploration of needed resources to expand the age inclusion. The PI was encouraged to expand their age range and offered resources to do so. Assistance included providing language for an IRB modification to expand the age of inclusion as appropriate and an offer of follow-up during study recruitment and retention to assist the study team in meeting the expanded age range of participants, among others. All qualitative information collected during these meetings was entered into the REDCap database and used for descriptive analysis. We identified the number of mismatched studies, the number of PIs accepting a meeting, and the number of studies that changed their protocol age range. The number of studies matching on protocol age from Phase 2 was compared to the number of studies matching on protocol age from Phase 1 to both disease demographics and disease prevalence using a proportion test.

Data collection for Phase 3 was similar to that of Phase 2 but focused on enrollment age. Studies in this phase were assessed on the last two criteria listed above: (3) how well the enrolled subject ages matched the disease demographics range and (4) how well the enrolled subject ages matched the disease prevalence. The number of studies that were a full match for each criterion were totaled separately, and the percentage was calculated for both. Study enrollment was reviewed every 6 months for eligible IRB studies, and our study team reached out to PIs whose study enrollment did not mirror demographics of the disease, offering support for inclusion enhancement and documenting responses. All qualitative information collected during these meetings was entered into the REDCap database and used for descriptive analysis. The number of mismatched studies, the number of PIs accepting a meeting, and the number of studies that changed their enrollment age were calculated. The number of studies matching on enrollment age from Phase 3 was compared to the number of studies matching on enrollment age from Phase 1 to both disease demographics and disease prevalence using a proportion test. R version 4.1.3 (R Foundation for Statistical Computing, Vienna, Austria) was used for all statistical analyses.

## Results

### Phase 1: Retrospective Review of Studies Closed to Enrollment before the NIH Lifespan Policy Went Into Effect

**Figure 3.**
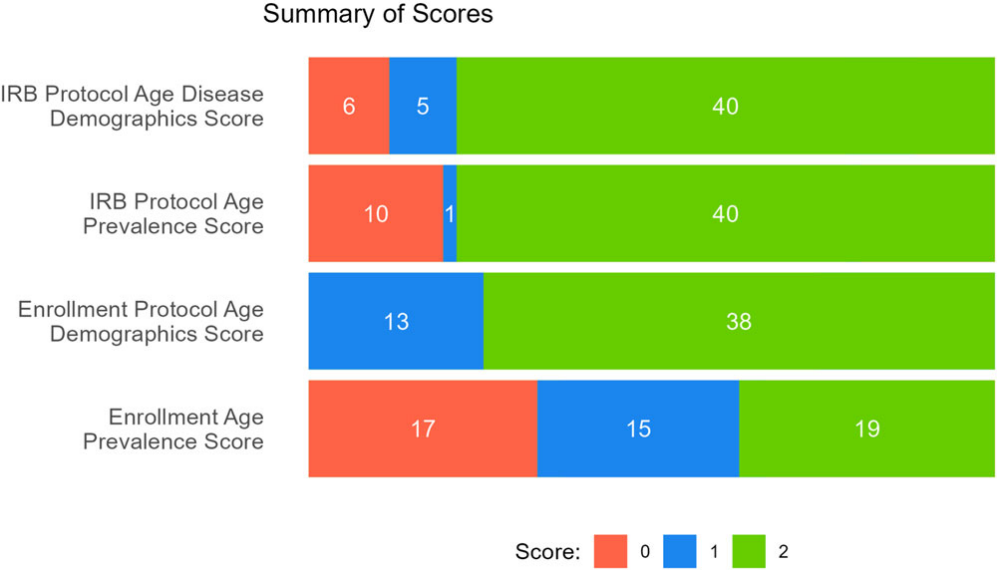
Summary of study scores during Phase 1 looking at IRB protocol age and enrollment age matching with disease demographics and prevalence.

After removing nine studies with no subjects enrolled and three studies for which disease demographics were not a good fit for our study of older adults (e.g., survival after childhood cancer), 51 studies remained in the analysis sample. The mean age of subjects included in the analysis was 58, with a median age of 63 years and a maximum age of 96 years. Most studies earned full points for IRB protocol ages matching disease demographics (40/51, 78%) and IRB protocol matching disease prevalence range (40/51, 78%), as well as for enrolling subjects across the disease demographic range (38/51, 74%) (Fig. 3). Lower scores were found for enrolling subjects within the disease prevalence range with 19/51 (37%) earning full points and 17/51 (33%) earning 0 points.

### Phase 2: Prospective Review of IRB Submissions for Compliance with the NIH Lifespan Policy

In Phase 2, 125 studies were eligible for the “IRB” phase (submitted to IRB between 1/1/2019 and 12/31/2020). Of the 125 eligible studies, 12% were NIH (10.4%) or partial NIH (1.6%) funded and subject to the NIH policy, 48% were “other” funding (defined as funding from a foundation, industry, or other sponsor), and 40% were unfunded/no funder listed. Eleven (8.8% of total N) studies meeting criteria for intervention in this phase were determined a “mismatch” to the disease demographics. Of the 11 meeting intervention criteria, 1 (9.1%) was NIH-funded and therefore subject to the NIH policy. Six (54.5%) were “other” funding, and four (36.4%) were unfunded/no funder listed. Of these 11 studies, 5 studies accepted the offer to meet, three studies changed their protocol age range post-intervention, and at the time of this writing, one study enrolled within the new higher/expanded protocol age range (Table 2).

**Table 2.** Outcomes of Phase 2 outreach to principal investigators

Study Code	Protocol age range pre-intervention (years)	Protocol age range post-intervention (years)	Change in protocol age range	Highest age of enrollment at last monitoring or when closed	Enrolled within new expanded age range – yes/no
I1	50–85	50–89	+ 4 years	76	No
I2	50–75	20–89	+ 14 years	80	Yes
I3	18–65	No change	No change	62	No
I4	40–85	No change	No change	80	No
I5	30–75	30–85	+ 10 years	74	No

### Phase 3: Prospective Review of Study Recruitment to Determine Compliance with the NIH Lifespan Policy

For Phase 3, eligibility included the 125 studies from Phase 2, plus 14 studies from the “gray zone,” defined as studies open to enrollment prior to 12/31/2018 that remained open past 1/1/2019 and thereby still enrolling when the NIH policy took effect. Of the 139 eligible studies, 13.7% were NIH (11.5%) or partial NIH (2.2%) funded and subject to the NIH policy, 50.4% were “other” funding, and 36% were unfunded/no funder listed. After removing 81 studies for incomplete or missing data, 58 studies remained in the analysis sample. Seven (12.1%) studies meeting criteria for intervention in this phase were determined a “mismatch” to the disease demographics. Of the seven meeting intervention criteria, none were NIH-funded and therefore subject to the NIH policy. Four (57.1%) were “other” funding, and three (42.9%) were unfunded/no funder listed. Of these seven studies, two studies accepted the offer to meet; however, none of the studies were able to change their upper age range enrollment post intervention.

### Overall

**Figure 4.**
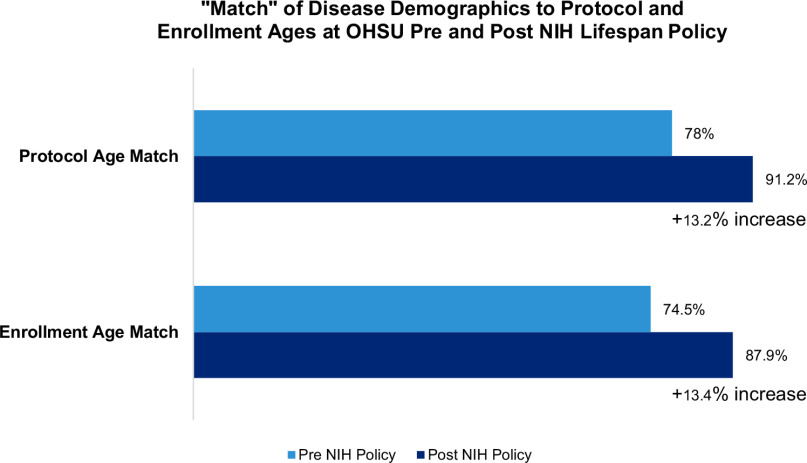
Comparison of age inclusion pre- and post-NIH Lifespan Policy.

For the prospective Phases 2 and 3, seven total studies accepted the offer to meet. Of those seven studies, three studies changed their protocol and enrollment ages as a result of our intervention. In comparing the protocol age ranges to the disease demographics, 91.2% of studies in Phase 2 were considered a “match” between these two. This is a significant 13.2% increase from the “match” of the same comparison in Phase 1 (78%, *p* = 0.04), potentially highlighting the natural influence of the NIH Lifespan Policy’s implementation (Fig. 4). Additionally, in Phase 3, 87.9% of studies were considered a “match” between disease demographics and enrolling ages, compared to 74.5% of studies in Phase 1 of this same comparison. This 13.4% increase between the pre- and post-NIH Lifespan phases was not statistically significant (*p* = 0.12).

## Discussion

Phase 1 of this study determined that while many investigators included older ages in their IRB protocol criteria and did enroll some subjects that fit the age range for disease demographics, a much smaller percentage of studies actually enrolled subjects in the upper ages of the disease prevalence. These results align with what is known currently about the exclusion of older subjects at the upper age ranges [1–3,7–9], but the first to clearly demonstrate that study enrollment frequently does not align with IRB protocol age ranges or disease demographics. The use of histograms to visually demonstrate study “mismatch” is a powerful way to demonstrate these discrepancies to study teams.

Phases 2 and 3 of this study determined that utilizing IRB and eCRIS records can assist institutional leadership in identifying gaps in study recruitment and retention. Once these gaps are identified, frameworks such as the “5T” model for inclusion of older adults can be utilized to provide opportunities for researcher training and protocol changes to enhance inclusion [10].

A major limitation to this study was the difficulty in finding and applying disease demographic ages and prevalence ranges [11]. Many disease demographic sources simply list “65+” or “75+,” rather than giving specific age ranges, making it challenging to ascertain demographic data for studies of diseases common in older adults. Clinicians often encounter diseases such as congestive heart failure, dementia, osteoporosis, etc., in patients in their 80s, 90s, and 100s. Research done on subjects in their 50s and 60s is less relevant to these age ranges, and lumping everyone “65+” together does not provide best practices for those who are much older. For our analysis, studies were included regardless of potential issues in the published disease demographics or prevalence ranges. A related limitation is that disease information across sources is inconsistent and sometimes contradictory. These limitations impacted our ability to be absolutely specific in determining if a study was a match, partial match, or mismatch; though even with this lack of specificity most investigators understood our overall goal and agreed to work toward inclusion of older adults in their research. A third limitation was the noncomprehensiveness of eCRIS reporting due to a decentralized research structure at OHSU. For example, only 51 of 63 studies in Phase 1 and only 58 of 139 studies in Phase 2 and 3 could be included due to incomplete or missing data. If this missing data was not random, it could have led to under- or over-reporting of inclusion gaps. A fourth limitation of this study is that numbers were small, and not all PIs responded to our outreach, so the impact of our outreach was not as robust as it might have been. Future work should seek to develop more strategies to encourage research teams to have inclusion be a priority for their studies.

In summary, this study developed tools and interventions to identify opportunities and assist research teams to enhance inclusion of older adults in research. The percentage of studies with IRB protocol ages and enrollment ages of subjects aligned with disease demographics increased after the implementation of the NIH Lifespan Policy. However, studies enrolling subjects within the disease prevalence range could be improved. Our outreach efforts shared strategies to enhance inclusion and resulted in raised awareness and changes to trial protocols. These strategies could be generalized to other underrepresented populations and could begin to truly ensure that the demographics of participants in research mirror the breadth of each disease and condition that is studied.

## Supplementary material

For supplementary material accompanying this paper visit https://doi.org/10.1017/cts.2023.539. click here to view supplementary material
