# Supplementary material for: Matching enrolled trial participants to disease demographics: Using IRB submissions to identify opportunities for researcher training
Source: J Clin Transl Sci. 2023 May 8;7(1):e114. doi: 10.1017/cts.2023.539 (PMC10260342; doi:10.1017/cts.2023.539)
Supplement: Supplementary file 1 [file S2059866123005393sup001.pdf]

Disease Type/Coding	Low Age	High Age	Prevalence Range (i.e. Mode)	Source
Alzheimer's Disease (AD) and other dementias	65	84+	75-84	Alzheimer's Association
Aneurysms/Stroke	45	75+	75+ (11% 75+)	Center's for Disease Control (CDC)
Arthritis	18	75+	45-64 (~60% <65yo) - CDC has another page that shows 55.4% of those 75+	Center's for Disease Control (CDC)
Atopic Dermatitis	<18	65+	0-5 (90% of AD get before age 5)	American Academy of Dermatology
Blood Disease/Leukemia	40	85+	80-90	Center's for Disease Control (CDC)
Brain Cancer	40	85+	75-84	Center's for Disease Control (CDC)
Breast Cancer	45	85+	70-79	Center's for Disease Control (CDC)
Cardiac Arrest	30	85+	60? (chart shows 80+, but report states most common at 60s)	American Heart Association
Cardiovascular Disease	45	75+	75+ (35% 75+)	Center's for Disease Control (CDC)
Chronic fatigue syndrome	20	60+	25-40	The Mayo Clinic
Chronic Obstructive Pulmonary Disease (COPD)	65	75+	65-74	Center's for Disease Control (CDC)
Colon cancer	40	85+	85+ (245.4 per 100K)	Center's for Disease Control (CDC)
Colonoscopy	45	85	45-75	American Cancer Society
Congenital Heart Disease	18	45+	45+	American Heart Association
Critical Care	18	85+	85+ (37%)	Society of Critical Care Medicine and Critical Care Medicine Journal
Diabetes (Type 1)	<18	70+	under 20	Center's for Disease Control (CDC) and Beyond Type 1
Diabetes (Type 2)	18	65+	65+ (25.2%) (Prevalence stated 65+, but new diagnosis shows >50% are 45-64 years)	Center's for Disease Control (CDC)
Endocrinology	25	85+	65-74	Journal of Clinical Endocrinology & Metabolism
Gastroenterology	<15	65+	65+ (14.62%)	NIH NIDDK
Head/Neck Cancer	45	85+	74-84	American Cancer Society, NIH Cancer Institute and the CDC
Heart Attack (Myocardial Infarction)	40	85+	75-84	American Heart Association
Heart Valve Disease	45	75+	75+ (13.3%)	The Heart Valve Society and The Lancet Journal
Hemophilia A	<18	65+	0-3 years	National Hemophilia Foundation and The CDC
Hernia	18	60+	41-60	The Mayo Clinic and NIH NIDKK
Herpesvirus	18	70+	35-45 (>50% have by age 40)	Center's for Disease Control (CDC)
Huntington Disease	30	80	30-50	National Institute of Health (NIH) and The Mayo Clinic
Hypertension	18	75+	65-74	Center's for Disease Control (CDC)
Kidney cancer	40	85+	70-79	Center's for Disease Control (CDC)
Lung Cancer	45	85+	75-84	Center's for Disease Control (CDC)
Multiple Sclerosis (MS)	20	50	30-35	National MS Society
Mycobacterium abcessus	18	85+	unknown/not found	Center's for Disease Control (CDC)
Nephrology	45	75+	75+ (6.8%)	Center's for Disease Control (CDC)
Neurodegeneration (non dementia, AD, PD)	30	85+	85+ (over 50%)	National Institute of Health (NIH) and The Ellison Medical Foundation
Oncology (Other)	18	70+	70+	American Cancer Society
Ophthalmology	55	80+	80+ (>14%)	The Journal of the American Medical Association and NIH Eye Institute
Orthopaedics	18	70+	45-64	Geriatric Orthopaedic Surgery & Rehabilitation Journal and American Academy of Orthopaedic Surgeons
Otolaryngology	18	65+	45-64	American Academy of Otolaryngology - Head and Neck Surgery
Pancreatic Cancer	50	85+	80-90	Center's for Disease Control (CDC)
Parkinson's Disease (PD)	45	85+	60-70 (also stated increased incident with 85+)	National Institute of Health (NIH) and The Parkinson's Foundation
Prostate Cancer	50	85+	65-75	Center's for Disease Control (CDC)
Pulmonology	35	70+	70+ (35%)	American Lung Association

Rheumatology	18	65+	65+ (>50%)	American College of Rheumatology and CDC
Thyroid Disease	18	85+	60-70 (risk increases with age)	The Mayo Clinic and American Thyroid Association
Tissue Cancer	40	60+	60+ (>50%)	American Cancer Society
Traumatic Brain Injury	<18	75+	75+	Center's for Disease Control (CDC)
Tumors	35	85+	65-74	NIH Cancer Institute
Urology	18	85+	51-60 (and 80+ dependent on specific disease)	NIH NIDDK
Von Willebrand Factor (Von Willebrand Disease)	<18	65+	5.-15	National Hemophilia Foundation and The CDC
